# Shaping the Future of Antimicrobial Therapy: Harnessing the Power of Antimicrobial Peptides in Biomedical Applications

**DOI:** 10.3390/jfb14110539

**Published:** 2023-11-02

**Authors:** Amit Kumar Tripathi, Jyotsana Singh, Rucha Trivedi, Payal Ranade

**Affiliations:** 1 Department of Microbiology, Immunology and Genetics, University of North Texas Health Science Center, Fort Worth, TX 76107, USA; ruchatrivedi@my.unthsc.edu (R.T.); payalranade@my.unthsc.edu (P.R.); 2 Hematopoietic Biology and Malignancy, The University of Texas MD Anderson Cancer Center, Houston, TX 77030, USA; jsingh1@mdanderson.org

**Keywords:** antimicrobial peptides, bio-films, biomedical applications, sepsis

## Abstract

Antimicrobial peptides (AMPs) have emerged as a promising class of bioactive molecules with the potential to combat infections associated with medical implants and biomaterials. This review article aims to provide a comprehensive analysis of the role of antimicrobial peptides in medical implants and biomaterials, along with their diverse clinical applications. The incorporation of AMPs into various medical implants and biomaterials has shown immense potential in mitigating biofilm formation and preventing implant-related infections. We review the latest advancements in biomedical sciences and discuss the AMPs that were immobilized successfully to enhance their efficacy and stability within the implant environment. We also highlight successful examples of AMP coatings for the treatment of surgical site infections (SSIs), contact lenses, dental applications, AMP-incorporated bone grafts, urinary tract infections (UTIs), medical implants, etc. Additionally, we discuss the potential challenges and prospects of AMPs in medical implants, such as effectiveness, instability and implant-related complications. We also discuss strategies that can be employed to overcome the limitations of AMP-coated biomaterials for prolonged longevity in clinical settings.

## 1. Introduction

Antimicrobial resistance (AMR) poses a significant global threat to public health, as it renders conventional antibiotics ineffective against bacterial infections [1]. The overuse and misuse of antibiotics have accelerated the emergence of drug-resistant strains, leading to increased morbidity, mortality, and healthcare costs. In the search for alternative therapeutic options, antimicrobial peptides (AMPs) have emerged as promising candidates due to their unique properties and ability to overcome cellular resistance mechanisms [2]. This resistance can be intrinsic, acquired through genetic mutations or horizontal gene transfer, and can spread rapidly within and between species [3]. The misuse of antibiotics in clinical and agricultural settings, inadequate infection control measures, and poor surveillance contribute to the proliferation of resistant strains [4]. The consequences of AMR are far-reaching and affect both individual patients and global healthcare systems [5]. Infections caused by drug-resistant pathogens are associated with higher rates of treatment failure, prolonged illness, and increased mortality. Additionally, the economic burden of AMR is substantial, with increased healthcare costs, extended hospital stays, and a reduction in productivity. If left unaddressed, AMR could potentially reverse the progress made in modern medicine, leading to a future where common infections become life-threatening once again. Another challenge is that there is an intricate relationship between AMR and some life-threatening diseases such as sepsis, highlighting the implications of AMR in sepsis management and the urgent need for comprehensive strategies to combat this dual crisis. Resistant bacteria, such as Methicillin-resistant Staphylococcus aureus (MRSA) or extended-spectrum beta-lactamase (ESBL)-producing *Enterobacteriaceae*, are common culprits in sepsis cases [6].

**Figure 1:**
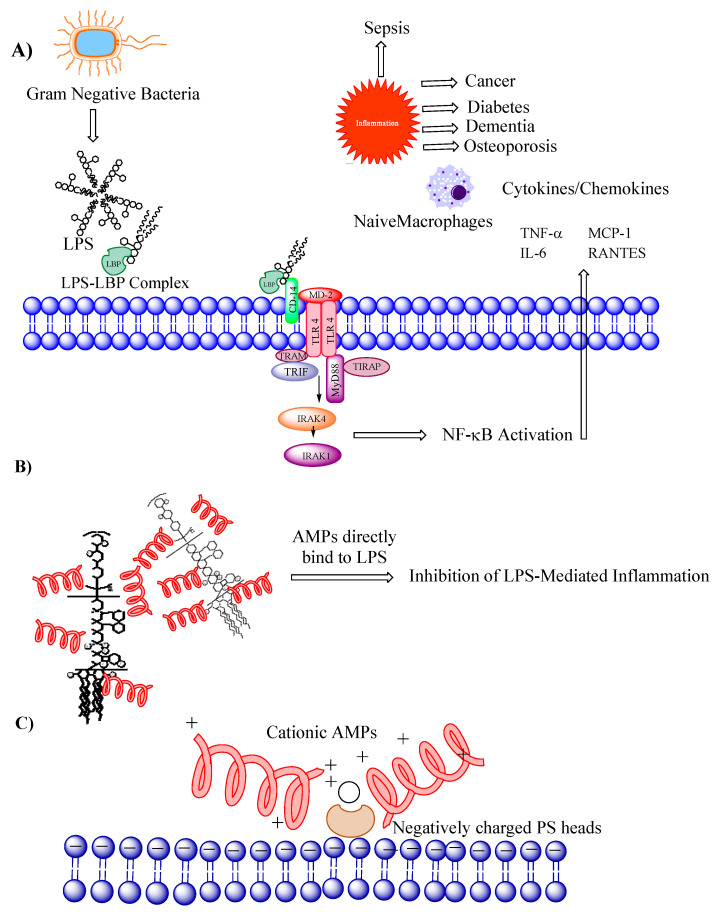
Multifaceted functions of (AMPs): (**A**) Depiction of the signaling pathway involved in Gram-negative bacteria-induced infection caused by lipopolysaccharides (LPS). (**B**) AMPs bind directly to LPS, leading to the inhibition of LPs-mediated inflammation. (**C**) Cationic AMPs bind to the negatively charged phosphatidyl serine (PS) heads on cancer cell membranes.

Sepsis is a critical condition that occurs as a result of an uncontrolled systemic inflammatory response to an infection. Lipopolysaccharide (LPS), also known as endotoxin, initially binds to the LPS binding protein (LBP). The LPS-LBP complex then binds to Toll-like receptor 4 (TLR4) and CD14, initiating a series of signaling events, leading to the activation of transcription factors, including nuclear factor-kappa B (NF-κB) [7]. Activation of these transcription factors results in the production and release of pro-inflammatory cytokines, such as tumor necrosis factor-alpha (TNF-α), interleukin-1 beta (IL-1β), and interleukin-6 (IL-6) [8,9,10] (Figure 1A).

AMPs hold promise as a potential treatment for sepsis as they not only possess direct antimicrobial activity but also exhibit anticancer and immunomodulatory properties [11]. They can modulate the immune response by enhancing the function of immune cells, promoting the clearance of pathogens, and regulating the release of pro-inflammatory cytokines. Many AMPs directly bind to LPS and inhibit downstream signaling [12] (Figure 1B). Interestingly, numerous antimicrobial peptides also interact with cancer cell membranes in a manner akin to their bacterial membrane targeting, owing to the anionic properties of cancer cell membranes [13] (Figure 1C).

The global market for peptide therapeutics is anticipated to reach a value of USD 44.43 billion by 2026, with a compound annual growth rate (CAGR) of 6.95% during the period from 2022 to 2026. Presently, over 80 peptide-based drugs are commercially available, addressing diverse health conditions such as cancer, osteoporosis, and diabetes [14]. Remarkably, the pipeline for peptide drugs is robust, with an estimated 400–600 peptides undergoing preclinical trials. This underscores the significant growth potential and expanding role of peptide therapeutics in addressing various medical challenges.

**Table 1:** Examples of Biomedical Applications of Antimicrobial Peptides.

Sl No	Biomedical Application	Peptide
1	Surgical site infection (SSI)	LL-37 [15], hBD2&3 [16], Protegrins [17], Histatins [18], Ranalexin [19], Pexiganan [20], Magainin [21], HNP1 [22]
2	Contact lens-associated microbial keratitis (CLMK)	α-MSH [23], Melimine [24], Pexiganan [25], Bacitracin [26], Dermcidin [27]
3	Dental applications	LL-37 [28], Dermaceptin [29], Nisin, Histatins [30], hBD1 [31], human beta-defensin-3 [32], human beta-defensin-5, Cateslytin [33], Myxinidin [34], HHC-36 [34]
4	Bone-graft applications	KLD [35], E14LKK [36]
5	Tissue generation	DermaceptinS4 [37], Thanatin [38], LLKKK18 [39], DPK-060 [40], SMAP-29 [41], G3KL [42], G3R, MSI-78
6	Anticancer agents	pAntp [43], KT2 [44], RT2 [45], LL37 [46], LTX-315, [46] melittin [47]

**Table 2:** Antimicrobial Peptide Names and Corresponding Sequences Discussed in the Review.

Sl. No	Peptide Name	Peptide Sequence	Reference	Clinical Tril ID (If Available) *
1	HNP-1	ACYCRIPACIAGERRYGTCIYQGRLWAFCC	[48]	
2	Drosocin	GKPRPYSPRPTSHPRPIRV	[49]	
3	Melittin	GIGAVLKVLTTGLPALISWIKRKRQQ	[47]	NCT02364349
4	LL-37	LLGDFFRKSKEKIGKEFKRIVQRIKDFLRNLVPRTES	[15]	NCT02225366
5	HBD-2	GIGDPVTCLKSGAICHPVFCPRRYKQIGTCGLPGTKCCKKP	[16]	
6	HBD-3	GIINTLQKYYCRVRGGRCAVLSCLPKEEQIGKCSTRGRKCCRRKK	[16]	
7	Protegrin-1	RGGRLCYCRRRFCVCVGR	[17]	
8	Ranalexin	FLGGLIKIVPAMICAVTKKC	[19]	
9	Pexiganan	GIGKFLKKAKKFGKAFVKILKK	[20]	NCT01594762
10	α-MSH	SYSMEHFRWGKPV	[23]	
11	Melimine	TLISWIKNKRKQRPRVSRRRRRRGGRRRR	[24]	
12	Magainin 2	GIGKFLHSAKKFGKAFVGEIMNS	[21]	NCT00563433
13	Dermcidin	SSLLEKGLDGAKKAVGGLGKLGKDAVEDLESVGKGAVHDVKDVLDSV	[27]	
14	Dermaceptin	ALWKTMLKKLGTMALHAGKAALGAAADTISQGTQ	[29]	
15	Nisin A	ITSISLCTPGCKTGALMGCNMKTATCHCSIHVSK	[36]	NCT02928042
16	Omiganan (Indolicidin derivative)	ILRWPWWPWRRK	[50,51]	NCT03071679
17	HBD-1	DHYNCVSSGGQCLYSACPIFTKIQGTCYRGKAKCCK	[31]	
18	HBD-5	GLDFSQPFPSGEFAVCESCKLGRGKCRKECLENEKPDGNCRLNFLCCRQRI	[52]	
19	Cateslytin	RSMRLSFRARGYGFR	[33]	
20	GH-12	GLLWHLLHHLLH	[53]	
21	Myxinidin	GIHDILKYGKPS	[54]	
22	HHC-36	KRWWKWWRR	[55]	
23	KLD-12	KLDLKLDLKLDL	[35]	
24	E14LKK	LKLLKKLLKLLKKL	[56]	
25	Dermaseptin-S4	ALWMTLLKKVLKAAAKAALNAVLVGANA	[57]	
26	Ib-AMP4	QWGRRCCGWGPGRRYCRRWC		
27	LLKKK18	KEFKRIVKRIKKFLRKLV	[39]	
28	DPK-060	GKHKNKGKKNGKHNGWKWWW	[40]	NCT01522391
29	SMAP-29	RGLRRLGRKIAHGVKKYGPTVLRIIRIAG	[37]	
30	MSI-78	GIGKFLKKAKKFGKAFVKILKK		NCT00563394
31	Bac2A	RLCRIVVIRVCR	[58]	
32	Chain201D	KWIVWRWRFKR	[59]	
33	E6	RRWRIVVIRVRRC	[60]	
34	Yao et al. (Unnamed Peptide)	(RWRWRWC–NH2)	[61]	
35	SESB2V	[(RGRKVVRR)2K]2KK	[62]	
36	Temporin-1CEa	FVDLKKIANIINSIF	[63]	
37	Esc(1–21)	GIFSKLAGKKIKNLLISGLKG-NH2	[64]	
38	18-mer LLKKK	KLFKRIVKRILKFLRKLV	[65]	
39	Thanatin	GSKKPVPIIYCNRRTGKCQRM	[38]	
40	Histatins	Sequence Differs Across Subtypes With Conserved Cationic Nature	[18,30]	
41	BmKn2	FIGAIARLLSKIFGKR	[66]	
42	Microcin E492	GETDPNTQLLNDLGNNMAWGAALGAPGGLGSAALGAAGGALQTVGQGLIDHGPVNVFIPVLIGPSWNGSGSGYNSATSSSGSGS	[67]	
43	BR2	RAGLQFPVGRLLRRLLR	[68]	
44	pAntp	RQIKIWFQNRRMKWKK	[69]	
45	pTAT	RKKRRQRRR	[70]	
46	KT2	NGVQPKYKWWKWWKKWW	[44]	
47	RT2	NGVQPKYRWWRWWRRWW	[45]	
48	LTX-315	KKWWKKWDip ** K	[71]	NCT04796194

* Source: Clinicaltrials.gov. ** Dip is β-diphenylalanine.

### 1.1. Discovery of AMPs

The discovery of AMPs has been a fascinating journey spanning several decades. It began with early observations of antimicrobial properties in natural substances used by ancient civilizations. In the 1980s, researchers stumbled upon a class of cationic peptides called defensins in rabbit leukocytes, marking the first identification of AMPs [72,73]. One of the earliest defensins to be discovered was human neutrophil peptide 1 (HNP-1), also known as alpha-defensin 1, which was identified in 1985 from human neutrophils [74]. Shortly after the discovery of HNP-1, several other defensins were identified in different organisms. For example, plant defensins were discovered in the late 1980s, and insect defensins were discovered in the early 1990s. Scientists then embarked on systematic screening efforts, exploring various sources including humans, animals, plants, and microorganisms to identify new AMPs. Advances in molecular biology techniques, such as cloning and DNA sequencing, allowed for the characterization of genes encoding AMPs and the production of large quantities of peptides for further study. Drosocin and melittin are examples of peptides that were identified using the above-mentioned approaches [75]. Drosocin was identified when researchers studying *Drosophila melanogaster* noticed that the flies had a robust immune response to bacterial infections [49]. They then extracted peptides from fly samples and later purified them using chromatography and mass spectrometry. The purified peptides were then subjected to further characterization to determine their chemical structure, activity, and mode of action [76,77]. A similar approach was followed for melittin as well [78]. After the confirmation of the antimicrobial and cytolytic activity in the bioassays, the active fraction was further purified using techniques such as high-performance liquid chromatography (HPLC) and mass spectrometry. The purified melittin was tested again for its antimicrobial, cytolytic, or other activities to confirm that it matched the initial observations and demonstrated the characteristic effects associated with melittin [79].

**Figure 2:**
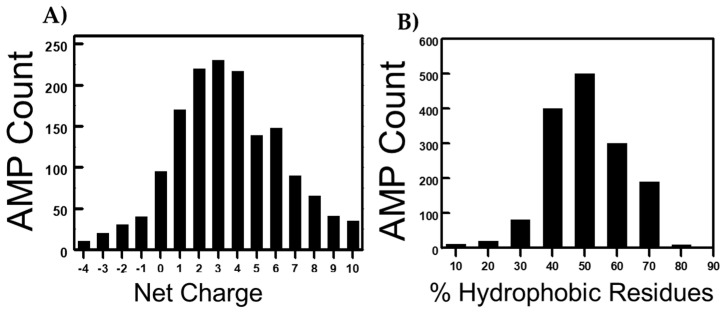
Distribution of antimicrobial peptides (AMPs) in the Antimicrobial Peptide Database concerning two critical parameters: net positive charge and percent hydrophobicity. Panel (**A**) provides insights into the distribution of AMPs according to their net charge, highlighting variations across different charge states. Panel (**B**) depicts the distribution based on the presence of hydrophobic amino acid residues within the AMPs. The Antimicrobial Peptide Database can be accessed at https://aps.unmc.edu/database (accessed on 15 June 2023) for comprehensive details.

Bioinformatics, genomics, and computational tools are also pivotal to predict and analyze peptide sequences with antimicrobial potential. Moreover, natural product peptide libraries and high-throughput screening methods have contributed to the discovery of novel AMPs as well. The latest in silico approaches to discovering AMPs include several computational methods that play a crucial role in the discovery of AMPs [80]. Researchers use various software tools and algorithms to predict and screen for potential antimicrobial peptides based on their sequence, structure, physicochemical properties, and known AMP databases. Examples of such tools include CAMPR3, AntiBP2, and CAMP2.0 [81,82]. In advanced machine learning techniques, deep learning and artificial neural networks are being increasingly employed to identify new AMPs [83]. These models are trained on large datasets of known AMPs and non-AMPs to recognize patterns and make predictions. Screening large libraries of peptides or peptide fragments is another strategy for identifying potential AMPs. Techniques like peptide arrays, phage display, and combinatorial chemistry are also commonly employed for this purpose. Other approaches include DNA sequencing technologies and structural biology approaches. In the former approach, the genetic information is analyzed, and by utilizing that knowledge, the researchers identify potential AMP-encoding genes and subsequently validate the antimicrobial activity of the corresponding peptides [84]. In structural biology approaches, rational designs of small α-helical peptides have been utilized to design AMPs with broad-spectrum activity against multidrug-resistant pathogens [85]. Techniques such as X-ray crystallography (X-ray), nuclear magnetic resonance (NMR) spectroscopy, and cryo-electron microscopy (cryo-EM) are used to elucidate the structures of AMPs and their interactions with microbial targets [86]. Antimicrobial and other bioactive peptides have also been derived from naturally occurring proteins and have been reported to possess both immunomodulatory and anticancer properties [87,88,89]. Table 1 and Table 2 present a comprehensive overview of all the discussed AMPs in this review. Various factors, such as net charge and hydrophobicity, govern the activity of AMPs [90]. The antimicrobial peptide database (https://aps.unmc.edu/database (accessed on 15th June 2023)) reveals that the majority of AMPs possess distinct levels of cationicity and hydrophobicity (Figure 2).

### 1.2. Harnessing Antimicrobial Peptides for Advanced Biomaterials

#### 1.2.1. Next-Level Surgical Innovation: Antimicrobial Peptide-Enhanced Sutures

Surgical site infections (SSIs) pose a significant challenge in modern healthcare, leading to increased patient morbidity, extended hospital stays, and substantial healthcare costs [91]. Antimicrobial peptides (AMPs) have emerged as a potential solution to combat SSIs due to their broad-spectrum antimicrobial activity and unique mechanisms of action. In recent years, researchers have explored the incorporation of AMPs into surgical sutures to create antimicrobial peptide-impregnated sutures (AMPIS). The incorporation of AMPs into sutures offers several advantages in the prevention of SSIs. Firstly, AMPs used in sutures are originally selected for their minimal cytotoxicity and immunogenicity, ensuring biocompatibility and compatibility with wound healing processes [92]. In addition, due to their broad-spectrum activity on drug-resistant strains, AMPs are suitable for combating polymicrobial infections. In addition to this, AMPs can disrupt biofilm formation, which gets embedded within a protective matrix [93]. By preventing biofilm formation, AMPIS can impede bacterial adhesion and subsequent colonization. This also reduces the infection rates. Studies have demonstrated that the use of AMPIS can significantly reduce the incidence of SSIs compared to traditional sutures.

LL-37 is a naturally occurring human cathelicidin that possesses broad-spectrum antimicrobial activity against bacteria, fungi, and viruses. It also exhibits immunomodulatory properties and promotes wound healing. Its incorporation into sutures can prevent surgical site infections [94]. Several members of the β-defensin family, such as HBD-2 and HBD-3, have been utilized in antimicrobial peptide-impregnated sutures. Other examples include the incorporation of Protegrin-1 and Histatins. While the former enhances the antimicrobial efficacy and helps prevent postoperative infections, the latter exhibits activity against various oral pathogens, including *Candida albicans*, *Streptococcus mutans*, and *Porphyromonas gingivalis.* The histatin-impregnated sutures are particularly useful in oral and maxillofacial surgeries [30,95]. Ranalexin is another antimicrobial peptide that was originally isolated from the skin secretions of the northern leopard frog. It possesses antimicrobial activity against various bacteria, including antibiotic-resistant strains. Ranalexin-based coatings have been investigated for their ability to inhibit wound and systemic MRSA infections [19].

Pexiganan is another example of an AMP that can be utilized for the incorporation of AMPs into sutures. It is a synthetic analog of the AMP magainin 2 and displays potent antimicrobial activity against both Gram-positive and Gram-negative bacteria, including MRSA. Pexiganan-infused collagen matrices have been shown to facilitate wound healing in rat models of infection [96]. Similarly, HNP-1-impregnated sutures have shown efficacy in preventing surgical site infections in various surgical procedures [97].

#### 1.2.2. Antimicrobial Peptide-Based Contact Lenses: The Future of Eye Care

**Figure 3:**
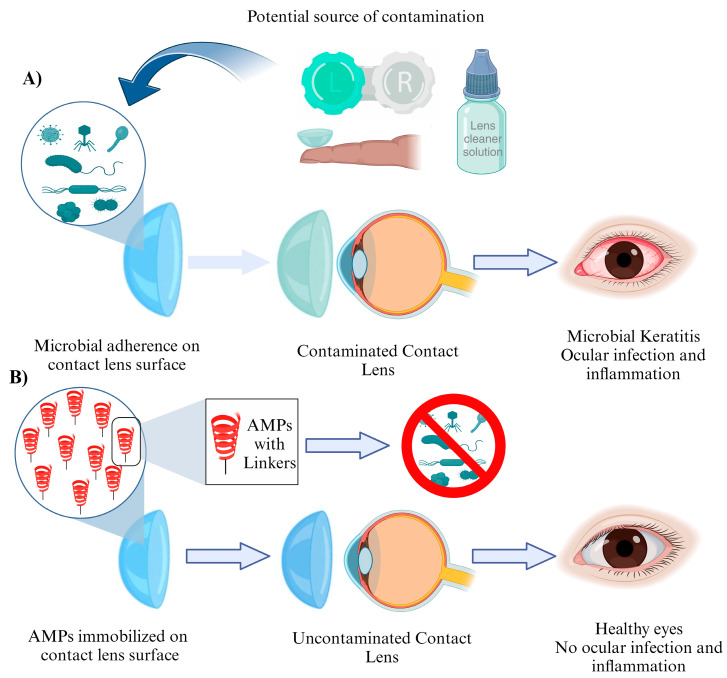
AMPs to fight against contact lens-associated microbial keratitis (CLMK). (**A**): Illustrates the process of microbial attachment to a contact lens, leading to microbial keratitis and eye infections. (**B**): Demonstrates the application of AMPs immobilized on contact lenses, effectively inhibiting microbial proliferation on the lens surface, thereby contributing to the maintenance of ocular health.

Contact lenses have revolutionized vision correction, providing a convenient and comfortable alternative to traditional eyeglasses. However, despite their numerous advantages, contact lenses pose a risk of infection due to microbial colonization on their surfaces [98]. This concern has led to significant efforts in developing novel contact lens materials with built-in antimicrobial properties. Contact lens-associated microbial keratitis (CLMK) is a serious condition that can lead to vision loss if left untreated [99]. The risk of CLMK arises from bacterial adhesion, biofilm formation, and subsequent infection on the lens surface. Traditional contact lenses, although effective in vision correction, lack inherent antimicrobial properties, making them susceptible to microbial colonization. Therefore, there is a pressing need for contact lenses that actively combat microbial growth to minimize the risk of infections. Researchers have focused on developing strategies to immobilize AMPs onto contact lens surfaces, ensuring sustained release and prolonged antimicrobial efficacy. One such approach involves the covalent attachment of AMPs to the lens material, allowing for controlled release over time. Another method utilizes hydrogels with AMPs encapsulated within, enabling a slow release of the peptide. These approaches not only provide antimicrobial properties but also maintain the biocompatibility and optical properties required for comfortable vision correction (Figure 3).

By inhibiting biofilm formation, AMPs prevent the accumulation of pathogens, ensuring a healthier lens surface. Furthermore, AMPs can combat multidrug-resistant microorganisms, which are becoming increasingly prevalent in healthcare settings.

Further advancements in AMP design, formulation, and delivery systems will undoubtedly enhance their antimicrobial efficacy and biocompatibility. Additionally, the integration of AMPs with other innovative technologies, such as smart materials or drug-delivery systems, may offer new possibilities for multifunctional contact lenses. As we move forward, extensive research, preclinical and clinical trials, and collaborations between scientists, engineers, and ophthalmologists are necessary to bring antimicrobial peptide-based contact lenses from the lab to the market. While challenges remain, ongoing research and technological advancements hold great promise for the development of safe and effective antimicrobial peptide-based contact lenses, benefiting countless individuals who rely on contact lenses for vision correction. α-MSH: α-melanocyte-stimulating hormone (α-MSH) is a naturally occurring peptide with immunomodulatory and antimicrobial properties. It has been incorporated into contact lenses to provide antimicrobial activity and promote ocular surface healing [100]. Another example of successful use of an AMPs in ocular health is temporins. Temporins are originally a group of antimicrobial peptides derived from the skin of frogs. Various temporin peptides have been investigated for their antimicrobial activity and have been incorporated into contact lenses to inhibit the growth of bacteria and fungi. Besides naturally occurring AMPs, there are certain synthetic peptides that were designed and synthesized and have been used for AMP-based contact lenses. A synthetic peptide melimine was produced by combining portions of the antimicrobial cationic peptides mellitin and protamine and has been studied for its potential in promoting wound healing and preventing infections when incorporated into contact lenses [101]. Pexiganan has been utilized in antimicrobial peptide-based contact lenses to enhance their antibacterial properties and prevent microbial colonization [102]. Apart from this, some cyclic peptide antibiotics such as Bacitracin have shown promise in their usage as antimicrobial formulations [103]. They have been used in contact lens solutions, due to their efficacy against Gram-positive bacteria. On similar lines, Dermcidin, an AMP found in human sweat has been explored for its potential in contact lens applications to prevent microbial contamination and infection [104].

#### 1.2.3. Antimicrobial Peptide-Conjugated Nanoparticles for Dental Applications: A Promising Approach for Combatting Oral Infections

**Figure 4:**
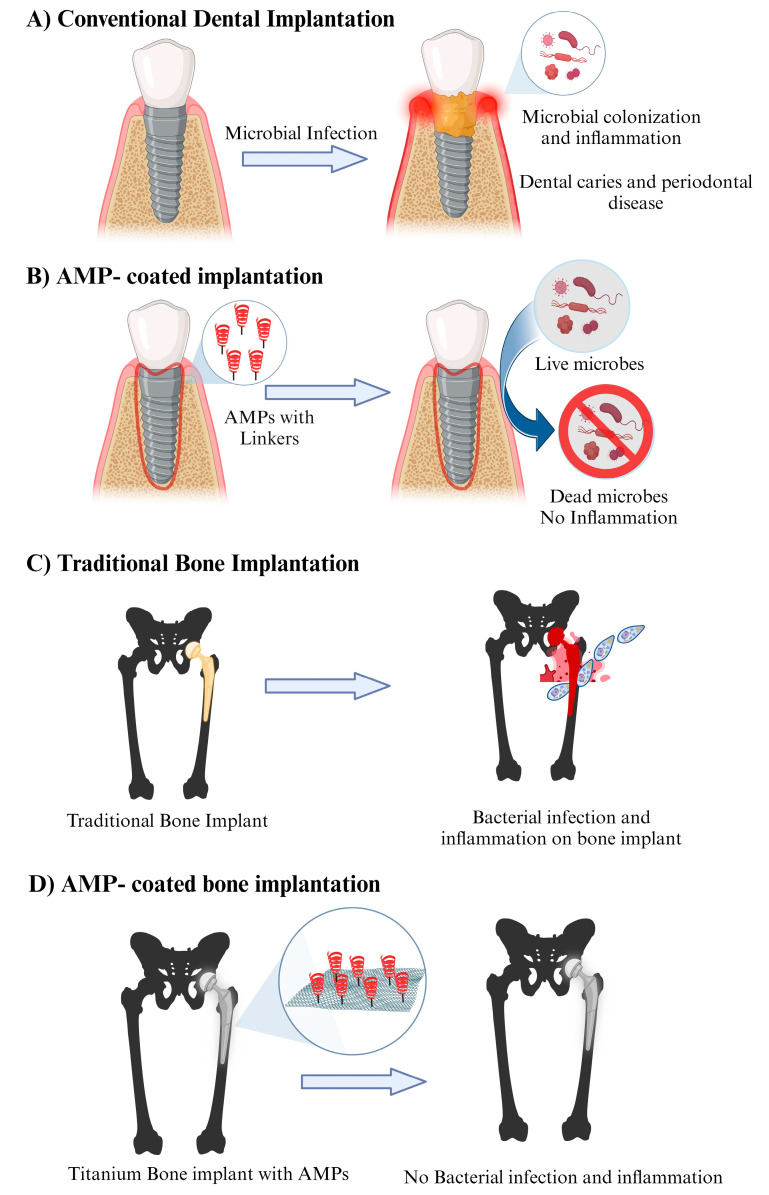
Applications of AMPs in dental care and bone graft treatment: (**A**) Conventional implants are unable to repel microbial infections; (**B**) AMP-coated implants kill bacteria promoting oral health; (**C**) Traditional bone implants lack inherent antibacterial properties; (**D**) Titanium alloy implants with AMPs inhibit bacterial growth.

Dentistry, as a branch of medicine, is no exception to antimicrobial resistance challenges [105]. Oral infections, such as dental caries and periodontal diseases, pose significant challenges to dental health worldwide. Conventional treatment methods often fall short in effectively targeting and eliminating microbial pathogens associated with these infections. In recent years, the development of antimicrobial peptide (AMP)-conjugated nanoparticles has emerged as a promising strategy for enhancing the antimicrobial efficacy and delivery of therapeutic agents in the field of dentistry [58]. The nanoparticle carrier enhances the antimicrobial efficacy of AMPs by increasing their local concentration at the site of infection, allowing for sustained release and reducing the required therapeutic dose. The nanoparticles can also facilitate the internalization of AMPs into microbial cells, leading to increased disruption of microbial membranes and inhibition of intracellular processes (Figure 4A,B). LL-37 has been investigated for its antimicrobial activity against oral pathogens and its potential application in dental materials, including composites [106]. Dermaseptins are a group of antimicrobial peptides found in the skin secretions of amphibians. They possess potent antimicrobial activity against a broad spectrum of microorganisms and have been utilized in dental applications as peptide-conjugated nanoparticles and fatty acids [29]. Nisin is a naturally occurring antimicrobial peptide produced by certain strains of *Lactococcus lactis.* It has potent antimicrobial activity against Gram-positive bacteria, including oral pathogens. Nisin has been conjugated to nanoparticles for dental applications to enhance their antimicrobial efficacy [107]. Indolicidin-coated silver nanoparticles have shown potent antibacterial activities in oral diseases [108]. The defensins viz. hBD-1, 3, 5 exert antibacterial activity against microbes involved in root canal infections, including *Enterococcus faecalis, Fusobacterium. nucleatum, Tannerella forsythia, Eikenella corrodens,* and *Candida albicans* [109,110,111]. Human β-defensin 3 (HBD3) peptide exhibits more antibacterial activity against mature multispecies biofilms containing *Actinomyces naeslundii, Ligilactobacillus salivarius,* and *Enterococcus faecalis* than either calcium hydroxide or 2% chlorhexidine solution [111].

Cateslytin is another AMP that is derived from the venom of the Brazilian scorpion *Tityus catesbeianus*. It exhibits antimicrobial activity against oral pathogens and can be used in dental applications as peptide-conjugated nanoparticles for its antibacterial effects [112]. GH-12 peptide polymers have been studied for the treatment of secondary caries and the enhanced durability of dental composite restorations [113]. Myxinidin is an antimicrobial peptide isolated from the mucus of hagfish. It exhibits potent antimicrobial activity against various bacteria and fungi including oral pathogens like *Pseudomonas aeruginosa* (Gram-negative bacteria) and *Candida albicans* [34]. Chen et al. demonstrated a method to construct antimicrobial titanium implants of HHC36 peptide that displayed remarkable antibacterial activity against both *Staphylococcus aureus* and *Escherichia coli* after only 2.5 h of incubation [114].

#### 1.2.4. Antimicrobial Peptide-Incorporated Bone Grafts: Revolutionizing Orthopedic Treatment

In the field of orthopedic surgery, bone grafts play a pivotal role in promoting bone regeneration and restoring skeletal integrity. However, the risk of postoperative infections remains a significant concern, leading to prolonged hospital stays, increased healthcare costs, and potential complications. To address this challenge, researchers have turned to AMPs as a promising solution. Bone grafts are widely used in orthopedic surgeries to repair fractures, promote bone healing, and reconstruct skeletal defects. They can be classified into autografts (taken from the patient’s own body), allografts (harvested from a donor of the same species), and synthetic grafts. While these grafts provide structural support and scaffolding for new bone formation, they are not inherently antimicrobial. Infection is a major concern following orthopedic surgeries, particularly in the context of bone grafts. Bacterial contamination can lead to implant-associated infections, which are challenging to treat due to the formation of biofilms and limited antibiotic penetration. Traditional strategies such as prophylactic antibiotic use have shown limitations, including antibiotic resistance and side effects. Therefore, there is a need for innovative approaches to combat infection. To harness the antimicrobial properties of AMPs, researchers have explored their incorporation into bone grafts. This involves either directly immobilizing AMPs onto graft surfaces or incorporating them into the graft matrix during the manufacturing process (Figure 4C,D). The goal is to create a localized, sustained release of AMPs to prevent infection while promoting bone healing. Incorporating AMPs into bone grafts offers several advantages. Firstly, it provides an immediate defense against potential pathogens at the surgical site, reducing the risk of infection. Secondly, AMPs have been shown to exhibit synergistic effects with conventional antibiotics, enhancing their efficacy and potentially reducing the required dosage. Thirdly, AMPs can aid in the promotion of bone regeneration by modulating inflammatory responses and enhancing angiogenesis. Several studies have demonstrated the efficacy of AMP-incorporated bone grafts in preclinical models. For example, one study showed that AMP-coated titanium implants reduced bacterial colonization and enhanced bone healing in a rat model [115]. As host cells and bacterial cells vie for control over the implant surface, the introduction of antimicrobial peptides onto the implant surfaces can tip the scales, thwarting implant infections. Yucesoy et al. pioneered a groundbreaking chimeric peptide designed to enhance the functionality of implant materials. This innovative peptide boasts a dual-purpose design, with one segment binding to the surface of a titanium alloy implant through a titanium-binding domain, while the other segment exhibits potent antimicrobial properties [116].

The KLD-12 peptide has also been studied for its bone-healing properties. It has been shown that the addition of arginine at the N-terminus converts it into an antimicrobial peptide [117]. Magainin-derived peptides such as E14LKK have also been investigated for their potential use in total-joint replacement prostheses [36]. Further research is needed to optimize AMP selection, develop reliable delivery systems, and evaluate long-term outcomes. Additionally, exploring the potential of AMPs in combination with other bioactive agents, such as growth factors or stem cells, could enhance bone regeneration and infection prevention synergistically.

#### 1.2.5. Antimicrobial Peptide-Based Scaffolds: Enhancing Tissue Regeneration with Antimicrobial Properties

Scaffolds play a crucial role in tissue engineering and regenerative medicine by providing a supportive framework for tissue regeneration. In recent years, the integration of antimicrobial peptides (AMPs) into scaffolds has emerged as a promising approach to enhance the antimicrobial properties of these constructs. By incorporating AMPs into scaffolds, researchers aim to prevent infections that may hinder successful tissue regeneration. The scaffolds serve as three-dimensional templates for cell attachment, proliferation, and differentiation in tissue engineering. They mimic the extracellular matrix (ECM) and provide structural support to regenerate damaged tissues. However, scaffolds alone are vulnerable to microbial colonization, which can lead to infections and impede the healing process. The incorporation of AMPs into scaffolds offers a synergistic approach by combining the regenerative capabilities of the scaffold with the antimicrobial properties of AMPs. Covalent attachment, physical adsorption, electrostatic interactions, and peptide amphiphiles are among the techniques utilized to ensure the retention and sustained release of AMPs from the scaffold structure. These strategies aim to maintain an effective concentration of AMPs at the scaffold–tissue interface, preventing microbial colonization without compromising cell viability or function. Antimicrobial peptide-based scaffolds offer several advantages over traditional scaffolds in tissue engineering. Firstly, they provide a controlled release of AMPs, ensuring a localized antimicrobial effect while minimizing systemic exposure. Secondly, AMPs can be tailored to exhibit selective antimicrobial activity, targeting specific pathogens without disrupting the commensal microbiota. Additionally, the incorporation of AMPs into scaffolds can facilitate faster wound healing, reduce the risk of post-operative infections, and enhance the success of tissue regeneration procedures. In skin tissue engineering, they have been employed to combat wound infections and promote re-epithelialization. Also, AMP-based scaffolds show potential in addressing chronic wounds, where infections can severely impede healing progress. Dermaseptin-S4 (S4) and its analogues have been studied for their potent antimicrobial activity and have been investigated as scaffolds for the development of novel AMP-based scaffolds [118]. Similarly, thanatin has been used as a scaffold for the development of peptide-based scaffolds with enhanced antimicrobial activity and stability [119]. Ib-AMPs (insect-derived antimicrobial peptides) are a diverse group of AMPs found in insects. Various ib-AMPs have been studied and used as scaffolds for the development of antimicrobial peptide-based scaffolds. LL-37 has also been used as a scaffold to design novel AMP-based scaffolds with enhanced antimicrobial activity and tissue injury healing [120]. LLKKK18, an engineered variant of the LL-37 peptide, has been designed to enhance its antimicrobial potency. It has been utilized in wound healing scaffolds for its broad-spectrum antimicrobial activity [39]. Similarly, DPK-060, a synthetic antimicrobial peptide, has been incorporated into wound healing scaffolds [121,122]. Another example of AMPs that are used in scaffolds is SMAP-29 (sheep myeloid antimicrobial peptide 29). SMAP-29, due to its strong antimicrobial activity against bacteria and fungi, can be used in wound healing scaffolds [122]. Abdel-Sayed et al. revealed the capacity of polycationic dendrimers to exert antiangiogenic effects on burn wounds. They synthesized peptide dendrimers using the amino acid residues L-lysine (G3KL) and L-arginine (G3R) distributed within the branches, resulting in two antimicrobial polycationic dendrimers (AMPDs). These AMPDs, specifically G3KL and G3R, were safely employed in combination with biological bandages comprising progenitor skin cells. This innovative approach effectively thwarted *Pseudomonas aeruginosa* infections and significantly enhanced wound healing in keratinocytes and endothelial cells [123]. MSI-78, a Magainin 2 analog, also possesses antimicrobial activity and has been used in wound healing scaffolds for its antimicrobial properties [124]. Wang and colleagues explored the formation of nanoparticles using MSI-78 and methoxy poly(ethylene glycol)-b-poly(α-glutamic acid). Their findings indicated a reduction in the hemolytic activity of the peptide on human red blood cells within the nanoparticle structure, while the peptide’s antibacterial effectiveness remained intact [125]. With continued advancements in this field, antimicrobial peptide-based scaffolds have the potential to revolutionize the field of tissue engineering, facilitating the development of safer and more effective therapeutic strategies for tissue regeneration.

#### 1.2.6. Antimicrobial Peptide-Coated Urinary Catheters: An Approach to Prevent Catheter-Associated Infections

**Figure 5:**
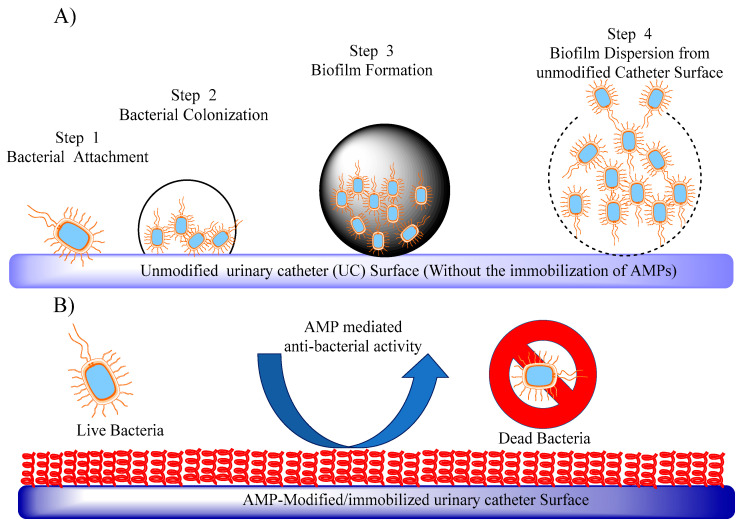
AMPs to fight catheter-associated urinary tract infections (CAUTIs). (**A**) Illustration depicting the initiation of bacterial colonization, biofilm development, and subsequent dispersion on an unmodified urinary catheter surface. (**B**) In contrast, representation displaying antibacterial activity on an AMP immobilized the urinary catheter surface.

Urinary tract infections (UTIs) are a prevalent healthcare-associated infection, and catheter-associated urinary tract infections (CAUTIs) contribute significantly to their occurrence [126]. UTIs are responsible for substantial morbidity, increased healthcare costs, and prolonged hospital stays. The emergence of antimicrobial resistance has limited the effectiveness of conventional antibiotics, necessitating the exploration of alternative strategies. CAUTIs are primarily caused by the introduction of bacteria into the urinary tract through the insertion of a catheter [127]. The longer the duration of catheterization, the greater the risk of infection. Several techniques have been developed to coat urinary catheters with AMPs, including physical adsorption, covalent binding, and incorporation into polymer coatings (Figure 5A,B). The choice of coating technique depends on factors such as stability, durability, and the release kinetics of AMPs. Li et al. demonstrated that arginine/lysine/tryptophan-rich antimicrobial peptides possess broad-spectrum antimicrobial properties and salt-tolerant characteristics on silicone surfaces, addressing the issue of catheter-associated urinary tract infections (CAUTIs) [128]. Through a process confirmed by X-ray photoelectron spectroscopy and water contact angle analyses, the researchers immobilized these peptides onto polydimethylsiloxane and urinary catheter surfaces using an allyl glycidyl ether (AGE) polymer brush interlayer. The resulting peptide-coated silicone surfaces exhibited remarkable antimicrobial efficacy against bacteria and fungi present in urine and phosphate-buffered saline solution. This effect was enhanced by the synergistic actions of the AGE polymer brush and AMPs, which not only prevented biofilm formation but also repelled cell adhesion. Importantly, the peptide-coated surface demonstrated no toxicity towards smooth muscle cells [128]. Bac2A is a synthetic antimicrobial peptide derived from the cathelicidin family. It has been evaluated for its antimicrobial efficacy and potential use in preventing biofilm formation on urinary catheters [129,130]. Monteiro et al. studied the peptide Chain201D (KWIVWRWRFKR) where they bound the peptide to ((1-mercapto-11-undecyl)-(tetra(ethylene glycol) (EG4)) terminated self-assembled monolayers (SAMs), (EG4-SAMs), activated by 1,1′-Carbonyldiimidazole (CDI) at different concentrations. The study showed the potential of utilizing the Chain201D peptide for the development of antimicrobial urinary catheters [59].

Yu et al. showed the potential of the E6 (RRWRIVVIRVRRC) peptide in treating CAUTIs. The E6 peptide, featuring a cysteine label at its C-terminus, was harnessed to coat polyurethane (PU) surfaces, revealing expansive antimicrobial capabilities [131]. This approach effectively thwarted catheter-associated infections in a mouse urinary infection model. Along similar lines, Yao et al. explored an AMP (RWRWRWC–NH2) and incorporated a thiol group into the cysteine residue, integrating it into a Cu^2+^-coordinated polydopamine coating on PU ureteral stents. The stents featuring the AMP coating demonstrated remarkable suppression of bacterial growth and biofilm formation, all while exhibiting negligible toxicity [61]. A synthetic peptide, CD4-PP, constructed through dimerization and backbone cyclization of the antimicrobial region from human cathelicidin LL-37, effectively inhibited uropathogens such as *Escherichia coli, Pseudomonas aeruginosa,* and *Klebsiella pneumoniae.* Additionally, catheter fragments coated with saline fluid enriched with CD4-PP showcased reduced *E. coli* attachment and dissolution of mature biofilm produced by these pathogens [132]. Thus, compared to traditional catheters, AMP-coated catheters have shown superior efficacy in reducing the risk of CAUTIs. They significantly inhibit bacterial adhesion, colonization, and biofilm formation, leading to a reduced incidence of infections. Antimicrobial peptide-coated urinary catheters hold great promise in preventing catheter-associated infections. Their unique mechanism of action, broad-spectrum activity, and low propensity for resistance make them an attractive alternative to conventional antibiotics. Despite challenges in selecting appropriate AMPs, overcoming resistance, and ensuring cost-effectiveness, the development and implementation of AMP-coated catheters offer significant potential to reduce the burden of CAUTIs and improve patient outcomes. Continued research and collaboration among scientists, clinicians, and regulatory authorities are crucial for the successful translation of this innovative technology into clinical practice. 

#### 1.2.7. Using Antimicrobial Peptides as Anticancer Agents

Cancer treatment remains a formidable challenge in global public health, characterized by a high mortality rate. Existing therapeutic approaches, encompassing surgery, radiotherapy, chemotherapy, or a combination thereof, aim to extend patient life expectancy. Antimicrobial peptides (AMPs) and anticancer peptides (ACPs) share common features, such as positive net charge, high hydrophobicity, and an amphipathic structure, enhancing their affinity for cell membranes. The similarities in characteristics prompt investigations into the antitumor activities of certain AMPs, potentially facilitating the improved design of ACPs. Notably, due to their distinct features, ACPs present a valuable resource with a reduced tendency for the development of cancer cell resistance, especially given the higher negative charge of cancer cell membranes. Various AMPs have demonstrated therapeutic efficacy against urinary bladder and colon cancers by influencing intracellular pathways, disrupting cell membranes, and directly inhibiting tumor cell proliferation. The scorpion-derived BmKn2 peptide exhibited cytotoxic effects against human colon cancer cells, while intratumoral administration of microcin E492 from *Klebsiella pneumonia* significantly reduced colorectal tumor cell mass in a zebrafish model. Combining AMPs with chemotherapeutic drugs, such as Gramicidin A and doxorubicin, displayed synergistic effects in colorectal cancer spheroids. Cell-penetrating peptides (CPPs), namely the BR2 peptide developed by Lim et al., exhibited noteworthy anticancer properties. In vitro experiments have demonstrated the BR2 peptide’s toxic effects on human cervical cancer and colon cancer cells. Furthermore, in vivo studies in a mice model of melanoma have underscored its anticancer efficacy. The CPPs typically consist of 30 or fewer amino acids, predominantly featuring positively charged amino acids such as lysine, arginine, or histidine. Notably, widely studied CPPs like pAntp and pTAT have found extensive application in delivering various therapeutic agents, including anticancer drugs, oligonucleotides, peptides, proteins, and viruses, into cells [69,70]. Similarly, KT2 [44] and RT2 [45] peptides, in combination with the chemotherapeutic drug 5-FU, exhibited enhanced efficacy against metastatic colon cancer cells. Clinical trials, like NCT02225366 and NCT01058616, have assessed the intratumoral administration of AMPs like LL37 and LTX-315, evaluating their antineoplastic effects and safety profiles. Notably, ongoing studies explore the combination of AMPs with immunotherapeutic agents, exemplified by the administration of LTX-315 with pembrolizumab in melanoma and triple-negative breast cancer (NCT04796194).

#### 1.2.8. The Hurdles Ahead: Constraints of Antimicrobial Peptide Biomaterials

While AMP-coated biomaterials offer promising solutions, it is important to recognize their limitations and challenges that may hinder their widespread use and effectiveness. Some AMP-coated biomaterials suffer from a limited spectrum of activity. 

When modifying the surface of medical implants, whether chemically (e.g., covalently) or physically, several problems can arise: in some cases, implant site infection due to bacterial colonization may lead to aseptic and septic loosening [133]. Ionization of the implant materials can cause a reaction with the biological host system, resulting in bone nonunion and implant loosening. Poor osseointegration can lead to implant failure, as the implant may not integrate well with the surrounding bone tissue [134]. Some surface modification techniques may increase the risk of inflammation, which can negatively affect the implant’s performance. Surface modification techniques can also lead to poor cell adhesion where metal implants, due to their smooth surfaces and high surface energy, create a poor environment for cell adhesion [135]. Additionally, the proteolytic enzymes present in biological fluids and tissues pose a challenge, as they can degrade AMPs, limiting their therapeutic potential. Temperature sensitivity is another critical factor, as AMPs may undergo conformational changes or lose their activity under extreme temperature conditions. Striking a delicate balance between these factors is essential for optimizing the performance of AMPs in therapeutic applications. When introduced into the bloodstream, AMPs may bind to various serum proteins, forming complexes that alter their pharmacokinetics and bioavailability. This interaction can lead to reduced concentrations of free, active AMPs, impacting their ability to combat microbial threats effectively.

More than 3000 AMPs have been reported and characterized, but in their natural state, most are not suitable as drugs for human medicine. FDA approvals are very limited due to the lack of cell selectivity, safety profile, and unexpected side effects. 

This limitation raises concerns regarding the reliability and long-term efficacy of AMP-coated biomaterials, as they may fail to protect against a wide range of microbial threats. Nisin, for example, exhibited potent activity against Gram-positive bacteria, including various strains of Staphylococcus and Streptococcus [136,137]. However, its effectiveness against Gram-negative bacteria is limited due to the outer membrane barrier possessed by these organisms [136]. Another major concern is with peptide stability and degradation. AMPs are susceptible to enzymatic degradation and can be inactivated by various factors present in the physiological environment. The instability of these peptides can significantly impact their antimicrobial activity and longevity on the biomaterial surface. Factors such as pH, temperature, and the presence of proteases can cause peptide degradation, leading to a decrease in their effectiveness over time [138,139]. This limitation poses challenges for maintaining sustained antimicrobial activity on the coated biomaterials throughout the intended duration of use. Several proline-rich peptides, such as Bac7 and Bac5, have shown antimicrobial activity. However, their stability and vulnerability to proteolytic degradation have posed challenges in developing AMP-coated biomaterials [140]. Biofilm formation is another common challenge associated with the use of biomaterials, and antimicrobial peptide coatings may not be effective in preventing or eradicating biofilms. While AMP coatings may have some impact on preventing initial bacterial adhesion, their effectiveness against established biofilms remains limited. Besides this, there are concerns about antimicrobial peptide-coated biomaterials having the potential for cytotoxicity and immunogenic reactions. Some AMPs can exhibit toxicity towards host cells, impairing the healing process and causing adverse effects on surrounding tissues. Additionally, the immune response to the presence of antimicrobial peptides may lead to inflammation or hypersensitivity reactions. Moreover, the lack of standardized testing methods and criteria for evaluating antimicrobial activity and long-term performance poses hurdles in assessing and comparing the effectiveness of different coatings. These regulatory challenges can delay the translation of antimicrobial peptide-coated biomaterials into clinical practice. Formulating stable and bioactive AMP-based formulations suitable for administration represents a significant hurdle, as issues like aggregation and loss of bioactivity during formulation, storage, and administration can compromise their efficacy. Furthermore, the diverse physicochemical properties of AMPs influence their interactions with delivery vehicles and biological systems, necessitating meticulous optimization to balance therapeutic potency with delivery efficiency. Achieving efficient penetration of AMPs through barriers such as tissues, cellular membranes, and biofilm matrices adds further complexity to their effective delivery.

#### 1.2.9. From Resistance to Resilience: Innovative Strategies to Overcome Limitations of Antimicrobial Peptide Coating Biomaterials

**Figure 6:**
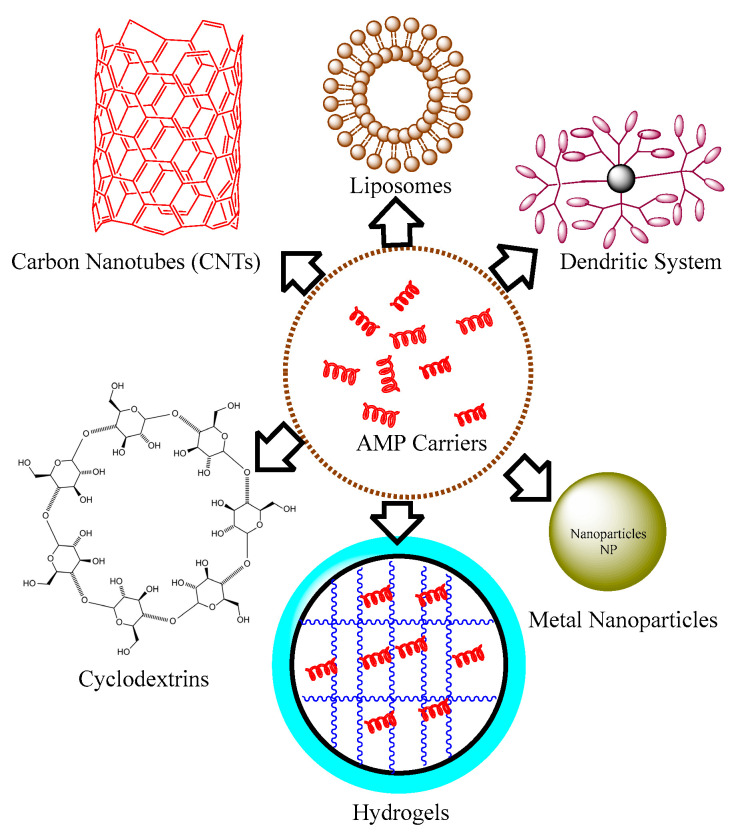
Diverse Nanocarriers for Encapsulation and Immobilization of Antimicrobial Peptides (AMPs).

The therapeutic efficacy of AMPs can be achieved through chemical modifications in constituent amino acids [141,142]. Some common strategies include incorporating D-amino acids instead of L-amino acids, introducing non-natural amino acids, or substituting specific amino acids known to be prone to degradation or enzymatic cleavage [143]. The sulfide bridges can provide structural stability to AMPs [144]. By introducing cysteine residues into the peptide sequence and allowing them to form disulfide bonds, the stability of the peptide can be enhanced. This method can be particularly effective for cyclic peptides. Cyclization is another chemical modification that reduces susceptibility to proteolysis and enhances resistance to chemical degradation [42]. Adding lipid moieties to AMPs can enhance their stability and membrane-binding properties. Lipidation can be achieved by attaching fatty acid chains or lipid-like groups to the peptide sequence, improving the peptide’s resistance to enzymatic degradation and increasing its overall amphipathicity [145]. The identification of new structural motifs present in AMPs can be used to immobilize them on the biomaterial surfaces. Introducing non-natural amino acids into the peptide sequence can enhance stability and resistance to proteolytic degradation. Unnatural amino acids with modified side chains or increased chemical stability can be utilized to improve peptide characteristics [146]. Some stabilizing agents can be incorporated into AMP formulations to enhance their stability. For example, protease inhibitors, metal chelators, or antioxidants can be added to protect AMPs from degradation by enzymes or reactive oxygen species [147]. Optimization of pharmacokinetic properties and the method validation of peptides can also help to prevent AMP-coating failures and degradation in clinical applications [148]. Materials with properties such as high surface area, stability, and compatibility should be used in coating applications. Examples of potential materials include metal oxides (e.g., silver, copper), carbon-based nanomaterials (e.g., graphene oxide), or polymer-based nanoparticles. Nanoparticles have effectively demonstrated their capacity to encapsulate established drugs used in the treatment of specific diseases, including certain types of cancer [149]. The interactions between the AMPs and chosen materials/nanoparticles should be well characterized and optimized (Figure 6). 

In 2017, Casciaro et al. documented the first report of a covalent linkage between the antimicrobial peptide esculentin-1a (which exhibits potent activity against *Pseudomonas aeruginosa* and soluble gold nanoparticles (AuNPs) via a polyethylene glycol (PEG) linker. The resultant compound, AuNPs@Esc(1-21), displayed nearly 15-fold increased antipseudomonal activity compared to Esc(1-21) alone, without inducing toxicity in human cells [150]. Chaudhari et al. conducted an assessment of the toxicity and antimicrobial efficacy of various antimicrobial peptides (TP359, TP226, and TP557) when incorporated onto silver-coated carbon nanotubes (CNTs). This investigation focused on combating *Staphylococcus aureus* infection within a three-dimensional human skin model of full thickness [151]. Nordström et al. showed that significant quantities of cationic antimicrobial peptides LL-37 and DPK-060 can be encapsulated within anionic poly(ethyl acrylate-co-methacrylic acid) microgels. These microgels demonstrated the ability to shield the enclosed peptides from degradation by infection-associated proteases, especially when a high microgel charge density was utilized [121]. Zetterberg et al. explored the application of PEG-stabilized liposomes as carriers for AMPs [152]. They examined the susceptibility of the melittin liposome to proteases and its antimicrobial efficacy in comparison to free melittin. Through repeated exposure to *Escherichia coli*, the melittin liposomes displayed notable bactericidal activity upon secondary exposure, outperforming free melittin. This effect was attributed to the time-dependent release of AMP from the liposomes. Interestingly, the liposome-encapsulated melittin was completely shielded from trypsin degradation, highlighting its enhanced stability [152]. Likewise, in various other investigations, cyclodextrins, hydrogels, and dendritic systems have been employed as carriers for antimicrobial peptides.

## 2. Conclusions

In conclusion, AMPs represent a promising avenue for combating infectious diseases and addressing the growing issue of antibiotic resistance. These small molecules, derived from natural sources, have shown potent antimicrobial activity against a wide range of pathogens, including bacteria, fungi, and even some viruses. By integrating AMPs onto the surface of biomaterials such as medical implants, wound dressings, or catheters, we can effectively inhibit the colonization and growth of harmful microorganisms, reducing the risk of infections. The use of AMP-coated biomaterials offers several advantages over traditional antimicrobial strategies. Furthermore, AMP-coated biomaterials have shown promising results in terms of biocompatibility and reduced cytotoxicity. Studies have demonstrated that AMPs can selectively kill pathogens while preserving host cells, minimizing the risk of adverse reactions or tissue damage. This makes them suitable for long-term use in medical devices and implants, where compatibility with the human body is crucial. Although there are still challenges to overcome, such as optimizing the stability and delivery of AMPs, as well as addressing potential manufacturing and regulatory hurdles, the development of AMP-coated biomaterials holds great promise. Continued research and innovation in this field will pave the way for new and effective strategies in combating infections and improving patient outcomes. In conclusion, antimicrobial peptides and their integration into AMP-coated biomaterials offer a compelling solution to the global challenge of antimicrobial resistance. Through further advancements and collaborations between researchers, healthcare professionals, and industry partners, we can harness the power of these naturally occurring molecules to develop innovative therapies and biomedical devices that effectively combat infections and enhance human health.

## Data Availability

The data supporting the findings of this study are available from the corresponding authors upon reasonable request.
